# Informal payments for modern family planning methods at public facilities in Tanzania: room for improvement

**DOI:** 10.1186/s12960-022-00712-5

**Published:** 2022-01-29

**Authors:** Clara E. Busse, Dickens Onyango, Katherine Tumlinson

**Affiliations:** 1 Carolina Population Center, University of North Carolina at Chapel Hill, NC Chapel Hill, United States of America; 2 Department of Maternal and Child Health, Gillings School of Global Public Health, University of North Carolina at Chapel Hill, NC Chapel Hill, United States of America; 3 Kisumu County Department of Health, P.O. BOX 3670, Kisumu, 40100 Kenya; 4 Utrecht University, Utrecht, The Netherlands; 5 Institute of Tropical Medicine, Antwerp, Belgium

**Keywords:** Informal payments, Informal fees, Contraception, Family planning, Quality-of-care, Access

## Abstract

**Background:**

Financial access to family planning (FP) is essential to the health and well-being of women in Tanzania. Tanzanian policy dictates that FP methods and services obtained at public facilities are provided for free. However, public sector FP is no longer free when providers solicit informal payments. In this analysis, we investigate the prevalence and amount of informal payments for FP in Tanzania.

**Methods:**

We used data from the 2015–2016 Tanzania Demographic and Health Survey to investigate whether informal payments for FP had been effectively eliminated by this policy.

**Results:**

We found that, at public sector facilities, the majority (84.6%) of women received their current FP method for free (95% confidence interval (CI): 81.9, 87.3), but this proportion varied meaningfully by facility and method type. Injectable contraception was the most commonly used method by women in the lowest wealth quintiles and was most frequently sought by these women from a government dispensary. One in four women (25.8%) seeking injectable contraception from government dispensaries reported paying a fee (95% CI: 19.5, 32.1). Among injectable users who reported payment for their current method, the mean cost at public sector facilities was 1420 Tanzanian Shillings (TSh) and the mean cost at private sector facilities was TSh 1930 (approximately 0.61 United States Dollars (USD) and 0.83 USD, respectively). Among implant users who reported payment for their current method, the mean cost at public sector facilities was TSh 4127 and the mean cost at private sector facilities was TSh 6194 (approximately 1.78 USD and 2.68 USD, respectively).

**Conclusion:**

These findings suggest that the majority of women visiting public facilities in Tanzania did not pay informal payments for FP methods or services; however, informal payments at public facilities did occur, varying by facility and method type. Adherence to existing policies mandating free FP methods and services at public facilities, especially government dispensaries, is critical for ensuring contraceptive access among the most economically vulnerable women.

## Background

Access to high-quality family planning (FP) services is essential for the empowerment of women and girls and an important strategy for the reduction of poverty and maternal and child mortality [1]. Effective, voluntary FP allows women and girls to prevent unintended pregnancies and space their births to protect their health and the health of their children. FP may also increase a woman’s autonomy in the home and improve her earning power and economic security [2]. Globally, 62% of married women ages 15 to 49 use a FP method, with 34% of this demographic using a FP method in low-income countries and 64% using a FP method in middle-income countries [3]. In sub-Saharan Africa, 34% of married women ages 15 to 49 use a FP method and 29% use a modern method [3]. Tanzania has a contraceptive prevalence rate among married women of 38% and there has been small but consistent rise in FP use in Tanzania over the last decade, yet disparities in FP use by sociodemographic characteristics make those who are least educated, poorest, and unmarried and sexually active especially vulnerable to unintended pregnancy [4, 5]. Thus, additional attention is needed to remove FP barriers for those women most vulnerable to the negative outcomes of an unintended pregnancy.

In Tanzania, known barriers to FP use include partner disapproval, limited healthcare access, myths and misinformation about FP, and concerns about costs of FP methods and services [6]. To ensure that cost is not a barrier to FP use, the Tanzanian Ministry of Health has mandated that FP services and methods are provided for free at all public facilities in Tanzania [6, 7]. At private sector facilities, clients may be charged a fee for the FP method and/or consultation [7]. However, research regarding informal payments made by FP clients in other sub-Saharan countries indicates that policies designed to ensure free contraceptive access do not always translate into practice. For example, despite policies enacted in 2013 that establish the provision of free FP at public facilities in Kenya, an analysis of 2014 Kenya Demographic and Health Survey (DHS) data by Radovich and colleagues found that only half of FP users who visited a public facility obtained their FP method for free at their most recent visit [8–11].

In fact, informal payments, defined as payments made by patients to healthcare providers that exceed the official cost of supplies or services, are common in a variety of healthcare settings in many low- and middle-income countries (LMICs) and informal payments often comprise a substantial share of healthcare spending in these contexts [12, 13]. Informal payments may take many forms, including charging for services that are supposed to be provided for free, pretending that there is a shortage of supplies and soliciting payments to obtain supplies, and requiring payments for higher quality care [13–17]. Informal payments may be solicited by health providers, but they may also be offered by patients as a demonstration of gratitude or as a method for receiving better care [16]. A study using focus groups of healthcare workers in Tanzania found that healthcare workers at all levels of care received informal payments through a variety of mechanisms [16]. Healthcare workers in this study reported that providers who gave their patients high-quality care were assumed to be receiving informal payments from them; for this reason, healthcare workers might be incentivized to reduce the quality of their health services to avoid being seen as corrupt [16]. Structural issues, such as low salaries of healthcare workers in public facilities, are thought to play a role in the system of informal payments in LMICs [14]. Despite their prevalence in LMICs broadly, the extent to which informal payments are leveraged for FP services is a gap in the current literature [18].

DHS and FP2020 data on the cost of current contraceptive methods are available for very few countries, but these data were collected in the Tanzania 2015–2016 DHS. Because informal payments for FP have the potential to prevent highly motivated women from obtaining their desired FP method, payments for FP at public sector facilities in Tanzania should be rigorously studied to ensure that existing policies are being effectively implemented.

To address this need, we used the Tanzania 2015–2016 DHS to describe the types and sources of FP methods and to characterize the prevalence and amount of informal payments. The Tanzania 2015–2016 DHS asked current users of an intrauterine device (IUD), injectable, implant, pill, and male condom, where they obtained their method, if they paid for their current FP method, and if so, how much. Using this existing data source, we investigated the extent to which informal payments in public sector facilities remain a barrier to FP provision in Tanzania. This paper is broadly modeled after Radovich and colleagues’ 2019 analysis to allow for comparison between Kenya and Tanzania [10].

## Methods

### Data source

We used data from the Tanzania DHS (2015–2016), a nationally representative, cross-sectional household survey with a two-stage cluster sampling design [19, 20]. Using this sampling strategy, 13,360 households were selected for the survey, of which 12,563 were occupied and completed the interview (98% response rate). In the interviewed households, 13,634 eligible women (age 15–49) were identified for individual interviews using the Woman’s Questionnaire, of which 13,266 women completed the interview (97% response rate). The Woman’s Questionnaire includes questions about the woman’s use of modern contraceptives, the source of the method, and the amount paid for it. The DHS report provides additional details about the sampling procedure, data collection, and specific interview questions [5].

We analyzed data for women who self-reported currently using a modern contraceptive [10]. Due to small sample sizes, we excluded those using male sterilization (*n* = 3), female condom (*n* = 2), emergency contraception (*n* = 3), the DHS FP method category “other modern method” (*n* = 1), and the Standard Days Method (*n* = 2).

### Variables

In Tanzania, FP is provided at a variety of facility types, which range in size from large hospitals, to medium-sized health centers, to small dispensaries, all of which are found in both the private and public sector. To capture this variation in facility types, we grouped women’s self-reported most recent source of modern FP method into seven categories: government hospital, government health center, government dispensary, private facility (including private hospital/clinic, private nursing/maternity home), non-governmental organization/faith-based facility, pharmacy/chemist, and other (including shop, mobile clinic, friend/relative, other, community health worker, community-based distributor, other private medical) [10]. We considered the first three categories to be public sector (government-provided services) and the latter four categories to be private sector [7, 10]. We excluded observations with missing data for most recent source of modern FP method (*n* = 37).

The Tanzania 2015–2016 DHS did not collect cost data for all types of methods. Therefore, to analyze informal payments, we limited this analysis to women who self-reported currently using a modern contraceptive method for which data on price were collected; this included the IUD, injectable, implant, pill, and male condom. Observations with missing values for informal payments were removed (*n* = 145). One woman responded with “do not know” for informal payments amount and was excluded. Self-reported payment values were inspected for improbable values. No values were found to be greater than 10 times the 95th percentile, the criterion specified for improbable values by Radovich and colleagues [10].

Several sociodemographic characteristics were included in our analysis. Quintile of household wealth was determined by the DHS based on measures of household assets appropriate for the Tanzanian context. Highest level of educational attainment was categorized as no education, primary, and secondary or higher. Due to sample size limitations, binary age was used, categorized as less than 30 years and 30 or more years.

### Analysis

Three main variables were of interest in our analysis: modern FP method, source of modern FP method, and payment for modern FP method. Payment for modern FP method was measured using two questions: (1) “Did you pay for (CURRENT METHOD)?”; (2) “How much did you pay for (CURRENT METHOD)?” [5]. We described modern contraceptive method mix by wealth quintile and source of FP. Injectable and implant use was reported by wealth quintile and source of FP. We then analyzed the proportion of modern contraceptive users reporting free FP planning by public sector facility type. Finally, we characterized the amount paid for current method among injectable and implant users who reported a non-zero payment for their current method at a public sector facility. Portions of our analysis (Fig. 3; Table 2) were limited to injectables and implants, as these were the only methods dispensed as a single unit and for which there was a sample size of at least 30 participants with non-zero payment for their current method, once stratified by facility type. Self-reported informal payments for FP method among injectable and implant users was reported in Tanzanian Shillings (TSh), with one USD being equal to approximately TSh 2300. Analyses were conducted in SAS version 9.4 (SAS Institute, Inc., Cary, North Carolina).

## Results

Modern contraceptive users with data for source of method available were included in our analytic sample (unweighted *n* = 3197). We found that the majority of women visiting public facilities in Tanzania did not pay informal payments for FP methods or services; however, informal payments at public facilities did occur, varying by facility and method type.

### Method mix

**Fig. 1:**
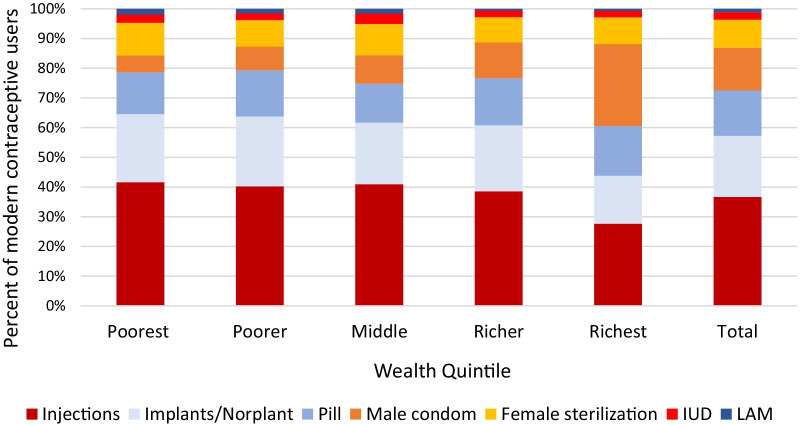
Method mix among modern contraceptive method users in Tanzania 2015–2016 Demographic Health Survey (unweighted *n* = 3187)

The mix of modern methods was fairly consistent across the four poorest wealth quintiles, with injections accounting for the largest share of use, followed by implants, and then the pill (Fig. 1). Male condoms increased in use with increasing wealth and accounted for less than 10% of use among the three poorest quintiles, but over one quarter of use among the richest. In the richest quintile, injectables were as common as male condoms and the pill and implants accounted for less than one fifth of methods each. Female sterilizations did not vary substantially by wealth quintile. Use of IUDs and the Lactational Amenorrhea Method (LAM) was uncommon across all wealth quintiles.

### Method source

**Fig. 2:**
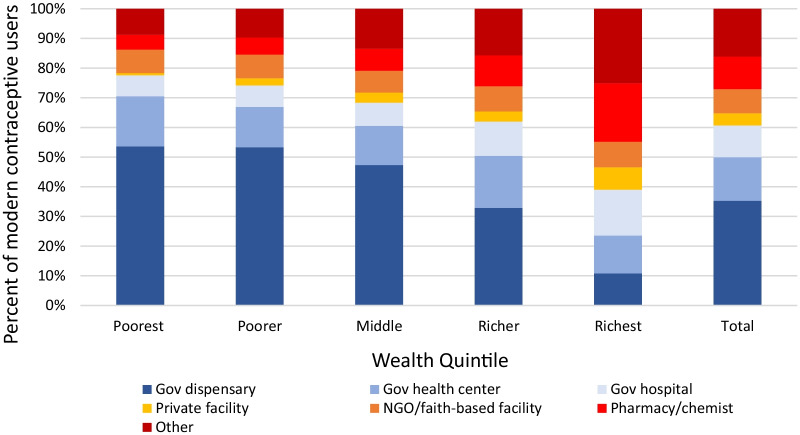
Facility type visited for family planning by wealth quintile among modern contraceptive method users in Tanzania 2015–2016 Demographic Health Survey (unweighted *n* = 3187)

Women sourced FP methods from a variety of facility types. Public facilities, including government hospitals, government health centers, and government dispensaries served as the source for the majority (ranging from approximately 60% in the richer quintile to over 75% in the poorest quintile) of current modern contraceptive methods for the four poorest wealth quintiles, but were the source for less than 40% of methods in the richest quintile (Fig. 2). Across all wealth quintiles, government dispensaries were the most common source of the current modern contraceptive method, serving as the source for one-third of current methods. Use of government dispensaries declined across wealth quintiles, ranging from the source of over half of current methods in the poorest quintile to approximately one-tenth in the richest quintile. Government health centers were the source for one-eighth of current methods in the total sample, which was relatively consistent across wealth quintiles. Use of private facilities increased with greater wealth, but served as the source for less than one-tenth of current methods in each wealth quintile. Pharmacies and the composite “other” category were most common in the richest quintile.

**Fig. 3:**
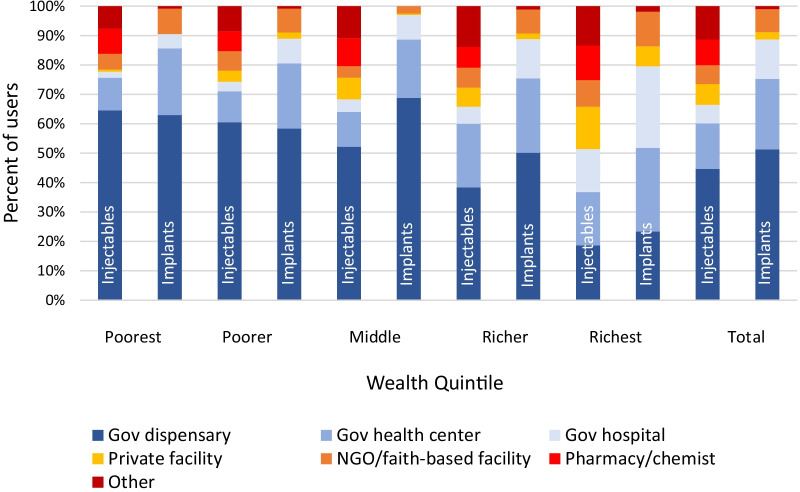
Provider type visited by wealth quintile among users of injectables and implants in Tanzania 2015–2016 Demographic Health Survey (unweighted *n* = 1857)

Government dispensaries were the most common source for injectable and implant users in the four poorest wealth quintiles, followed by government health centers (Fig. 3). Injectable and implant users in the richest quintile had the broadest range of sources, with each source accounting for less than one-third of all sources for the current method.

### Free family planning at public sector facilities

Current users of the IUD, injectable, implant, pill and male condom reported whether they paid for their current method, and if so, how much. Averaged across the three types of public facilities, 85% of FP users who visited a public facility reported that they received free FP (Table 1). This proportion varied across public facility type, with government hospitals reported to provide the greatest proportion of free FP (94%), followed by government dispensaries (84%), and government health centers (83%). Free FP provision varied by method type, ranging from 78% of injectable users and 89% of pill users reporting free FP to 98% of IUD users reporting free FP at all public sector facilities. Across wealth quintiles, education, and age, the proportion reporting free FP was high, with no discernable pattern suggesting an association between any of these characteristics and the likelihood of being asked to pay for FP.

**Table 1:** Proportion of modern contraceptive method users reporting free family planning by public sector provider type

	Government hospital (unweighted *n* = 214; weighted *n* = 220.3)	Government health center (unweighted *n* = 424; weighted *n* = 440.7)	Government dispensary (unweighted *n* = 1141; weighted *n* = 1174.0)	Total public (unweighted *n* = 1779 weighted *n* = 1835.0)
Overall (95% CI)	93.7 (90.3, 97.2)	82.9 (78.1, 87.7)	83.5 (79.9, 87.2)	84.6 (81.9, 87.3)
Modern method				
Injectable	92.6 (86.4, 98.8)	81.7 (74.2, 89.3)	74.2 (67.9, 80.5)	77.6 (72.7, 82.5)
Implant	93.4 (88.4, 98.4)	80.3 (72.8, 87.8)	94.1 (90.7, 97.6)	90.3 (87.3, 93.2)
Pill	92.9 (79.3, 100.0)	91.2 (80.1, 100.0)	87.8 (81.7, 93.8)	89.0 (84.0, 94.0)
Condom	100.0 (100.0, 100.0)	100.0 (100.0, 100.0)	90.2 (78.6, 100.0)	93.9 (86.5, 100.0)
IUD	100.0 (100.0, 100.0)	94.5 (86.4, 100.0)	100.0 (100.0, 100.0)	98.0 (95.1, 100.0)
Wealth quintile				
Poorest	100.0 (100.0, 100.0)	80.5 (67.0, 94.0)	81.2 (72.8, 89.6)	81.7 (78.8, 88.7)
Poorer	90.4 (78.1, 100.0)	94.6 (89.5, 99.8)	87.1 (81.9, 92.3)	88.6 (84.3, 92.9)
Middle	95.7 (89.6, 100.0)	79.5 (65.8, 93.3)	78.2 (72.1, 84.3)	79.8 (74.3, 85.3)
Richer	91.5 (82.4, 100.0)	85.9 (78.3, 93.5)	86.2 (81.1, 91.3)	86.8 (83.0, 90.5)
Richest	94.6 (89.7, 99.5)	76.0 (65.2, 87.7)	88.1 (79.9, 96.3)	85.7 (80.8, 90.6)
Educational attainment				
No education	87.1 (69.3, 100.0)	84.5 (72.1, 96.9)	78.2 (70.7, 85.7)	80.1 (73.9, 86.2)
Primary	93.4 (88.5, 98.3)	86.0 (80.8, 91.1)	84.4 (80.6, 88.3)	85.7 (82.7, 88.7)
Secondary or higher	96.4 (92.2, 100.0)	69.1 (54.8, 83.5)	85.6 (78.5, 92.7)	83.8 (78.2, 89.5)
Age group (years)				
< 30	94.0 (89.6, 98.4)	78.3 (70.4, 86.2)	83.0 (78.2, 87.7)	83.3 (79.6, 86.9)
30+	93.3 (87.5, 99.2)	88.0 (81.7, 94.2)	84.1 (80.0, 88.4)	86.1 (82.8, 89.3)

*IUD* intrauterine device

The proportion of method users reporting free FP was lowest among injectable users who visited government dispensaries; there, just three out of four women reported free FP (74%). At government dispensaries, approximately nine out of ten pill users and condom users reported free FP (88% and 90%, respectively). Similarly, only eight out of ten injectable and implant users reported free FP at government health centers (82% and 80%, respectively).

### Payment for injectable and implant at public and private sector facilities

As shown in Table 1, 78% of injectable users and 90% of implant users who visited a public sector facility reported free FP. Among injectable users reporting non-zero payment for their current method, the mean cost for an injectable at a public sector facility was TSh 1420 (approximately 0.61 USD), and at a private sector facility the mean cost was TSh 1930 (approximately 0.83 USD) (Table 2). Among implant users reporting non-zero payment for their current method, the mean cost for an implant at a public sector facility was TSh 4127 (approximately 1.78 USD), and at a private sector facility the mean cost was TSh 6194 (approximately 2.68 USD). Small sample sizes prevented the analysis of cost by sociodemographic characteristics.

**Table 2:** Amount paid for current contraceptive method among injectable and implant users who reported non-zero payment

	Total public sector	Total private sector
*Injectable*		
*N*	157	229
Mean cost	1420 (TSh)	1930 (TSh)
Minimum	100 (TSh)	100 (TSh)
25th percentile	414 (TSh)	1347 (TSh)
Median	1329 (TSh)	1690 (TSh)
75th percentile	1855 (TSh)	1935 (TSh)
Maximum	1500 (TSh)	10,000 (TSh)
*Implant*		
*N*	55	31
Mean cost	4127 (TSh)	6194 (TSh)
Minimum	500 (TSh)	1000 (TSh)
25th percentile	1758 (TSh)	2688 (TSh)
Median	2406 (TSh)	4250 (TSh)
75th percentile	3850 (TSh)	7536 (TSh)
Maximum	20,000 (TSh)	22,000 (TSh)

*TSh* Tanzanian Shilling

## Discussion

In our analysis of 2015–2016 Tanzania DHS data, we found that the vast majority of modern FP users, approximately 85%, reported free FP at public sector facilities. In the four poorest wealth quintiles, public facilities (government hospitals, government health centers, and government dispensaries) were the source for the majority of current contraceptive methods. In all but the richest wealth quintile, injections and implants were the most commonly used modern FP methods. These findings suggest that most providers within public sector facilities in Tanzania are adhering to enacted policies, which stipulate that FP methods and services should be provided for free at public facilities [6, 7]. These findings contrast with those presented by Radovich and colleagues in their analysis of 2014 Kenya DHS data, in which they found that only half of public sector FP users reported that they obtained their method for free despite policies stating that FP methods should be provided for free at public sector facilities [10].

However, implementation of this important policy can still be improved as we found that 22% of injectable users and 10% of implant users who visited public facilities reported payment for their current method at a public sector facility. At government dispensaries, one out of every six women reported informal payments for FP methods and/or services. Furthermore, one in every four women who obtained an injectable at a government dispensary was asked for informal payments. These findings are especially meaningful, because government dispensaries are the most popular source and implants and injectables are the most popular methods among women in the lowest wealth quintile.

It may be that informal payments arise, because providers are targeting particular patients (e.g., women with low socioeconomic status); however, it may also be that providers at particular facility types are more likely to leverage informal payments, regardless of patient demographics; for example, providers working at government dispensaries may be more likely to be located in a remote area with limited supervision and/or be the sole healthcare provider at their facility, both of which may facilitate solicitation of unsanctioned informal payments. Delineating between these possibilities is outside the scope of the present analysis, but should be a focus of future work examining the etiology of informal payments for FP.

The informal payments reported for injectables at public facilities may be prohibitive for women living around and below the poverty line. In 2018, 14 million people lived below the Tanzanian poverty line of TSh 49,320 per adult per month (about 21 USD) and 26 million (approximately 49% of the population in Tanzania) lived below the international poverty line of 1.90 USD per person per day [21]. Among those currently using injectables who paid for their method at a public sector facility, the mean cost was TSh 1420, or approximately 0.61 USD. Though this cost may seem trivial to people not living in poverty, for millions of women in Tanzania, an informal payment of 0.61 USD for an injectable would be a challenging sum to procure.

Demand for and use of implants has increased rapidly in many countries in sub-Saharan Africa over the last decade [22]. In this analysis, we found that, among those who paid for their current method at a public sector facility, the mean informal payment for implants was TSh 4127 (approximately 1.78 USD), which is 3 times the mean informal payment for injectables among those who reported paying any amount for FP at a public facility. This informal payment may be prohibitive for many women. Thus, it is possible that informal payments for implants might be suppressing the true demand for this highly effective method in Tanzania.

Provider demand of informal payments for perinatal healthcare is common in many countries around the world and some studies have focused on their impact on sexual and reproductive health services [10, 12, 23–30]. The frequency of informal payments differs substantially by country, ranging from 3% in Peru to 96% in Pakistan [12]. While informal payments are quite common across South Asian countries, payment frequency varies widely by country in East Asia, Latin America, and Eastern Europe; in sub-Saharan Africa, evidence from studies in Uganda, Mozambique and Ethiopia suggests that informal payments to public sector providers are common [12]. The size of these informal payments varies, but can add up to a formidable sum, especially for patients with low socioeconomic status and for those who are charged informal payments at several points while accessing care. Tragically, knowledge that providers may solicit informal payments when patients seek care may prevent care seeking altogether, particularly for patients who have few financial resources [23, 25, 29, 30].

The literature suggests several reasons for informal payments in public sector facilities, which may be relevant to the Tanzanian context. Tumlinson and colleagues analyzed in-depth semi-structured interviews with 20 public and private sector reproductive healthcare workers in Kenya, and found that providers cited low public sector wages as a chief reason that providers ask patients for informal payments [18]. Providers reported that senior staff often worked together to solicit informal payments and explained that patients might be charged informal payments, because they do not know which services should be provided free of charge. These findings suggest both individual-level and structural-level levers for reducing the solicitation of informal payments at public sector facilities, including educating patients about the free provision of FP at public facilities in Tanzania and fairly compensating public sector healthcare workers for their labor. Salaries of providers in public sector facilities in Tanzania are typically standardized within specific job “groups,” which, in theory, are based on education and years of experience (though, in practice, promotions can be delayed). In addition, wages may fail to keep pace with inflation and there may be variation across facilities or regions with regard to timely payment of wages (In an email from D. Onyango, MD in January 2022).

There is limited evidence evaluating strategies to reduce informal payments for family planning and reproductive health services, though social accountability approaches have emerged as promising strategies [31, 32]. One social accountability approach, called the Community Score Card, was implemented in Malawi and found to increase client satisfaction, contraceptive use, and service delivery compared to communities who did not receive the intervention, but the intervention’s impact on informal payments was not assessed [33]. Another social accountability intervention was designed to decrease demand for informal payments for maternal health care and implemented in India. The intervention appeared to increase the empowerment and knowledge of participating community members and anecdotal evidence suggested a decrease in demands for informal payments [17, 34].

Though informal payments may be less of a problem in Tanzania than in neighboring countries, progress is still needed to eliminate all informal fees at public sector facilities in Tanzania. Further research should investigate the reasons that informal payments are solicited in public sector facilities, especially government dispensaries, and identify methods for reducing these fees to ensure that the most vulnerable populations have access to the FP services needed to achieve their reproductive goals.

### Strengths

This study uses nationally representative data to investigate informal payments for FP in Tanzania. We use existing data to study an important topic with critical implications for FP access.

### Limitations

Our investigation of informal payments for modern contraceptives was limited by sample size constraints. Because a minority of women reported paying any amount at public or private facilities for implants or injectables, we were not able to assess whether payment amount varied by sociodemographic factors, such as education, age, or wealth. In addition, these existing data do not allow us to assess the proportion of women who were unable to pay informal payments and, therefore, left the facility without a method of contraception. Given evidence suggesting women with fewer resources may be more likely to be asked for informal payments [17], new indicators are needed to track this equity concern. We are also unable to ascertain whether some FP clients were able to avoid informal payments by insisting on free services or switching to a different provider or different public sector facility. These are important areas for future research.

## Conclusion

Voluntary, safe family planning is critical for the health of women and children. Tanzania’s contraceptive prevalence rate among married women is just 38% and disparities in FP use make women who are least educated, poorest, and unmarried and sexually active particularly vulnerable to unintended pregnancy [3]. Ensuring access to FP for all who want it requires the elimination of barriers to FP, and concerns about costs of FP are a known barrier [6]. Tanzanian policies dictate that FP methods and services are provided for free at public sector facilities, but to our knowledge, no published studies investigate the implementation of this policy [6, 7]. Our findings indicate that most modern FP users report receiving their method for free at public sector facilities in Tanzania. Still, some implant and injectable users report informal payments for their methods at public facilities, suggesting that implementation of FP cost initiatives in Tanzania could be improved. Future research should investigate whether informal payments prevent women from obtaining their desired FP method and characterize how informal payments are leveraged at government dispensaries and health centers to inform interventions.

## Data Availability

The Demographic and Health Survey data used in this study are available from the Demographic and Health Survey Program, [https://dhsprogram.com/data/].

## Abbreviations

FP: Family planning
CI: Confidence interval
TSh: Tanzanian Shillings
USD: United States Dollars
DHS: Demographic Health Survey
LMICs: Low- and middle-income countries
IUD: Intrauterine device
LAM: Lactational Amenorrhea Method
